# Cediranib Induces Apoptosis, G1 Phase Cell Cycle Arrest, and Autophagy in Non-Small-Cell Lung Cancer Cell A549 In Vitro

**DOI:** 10.1155/2021/5582648

**Published:** 2021-03-30

**Authors:** Menghuan Guo, Zhiyuan Liu, Jing Si, Jinhua Zhang, Jin Zhao, Zhong Guo, Yi Xie, Hong Zhang, Lu Gan

**Affiliations:** ^1^School of Pharmacy, Lanzhou University, Lanzhou, Gansu 730000, China; ^2^Advanced Energy Science and Technology Guangdong Laboratory, Huizhou, Guangdong 516029, China; ^3^Gansu Key Laboratory of Environmental Friendly Composites and Biomass Utilization, College of Chemical Engineering, Northwest Minzu University, Lanzhou, Gansu 730030, China; ^4^Institute of Modern Physics, Chinese Academy of Sciences, Lanzhou 730000, China; ^5^Medical College of Northwest Minzu University, Lanzhou, Gansu 730030, China

## Abstract

Lung cancer remains the leading cause of cancer death worldwide. Late diagnosis, chemoresistance, and metastasis are the main reasons for the high mortality rate of lung cancer. Therefore, the development of other treatments is urgent. Cediranib (CED), a vascular endothelial growth factor receptor (VEGFR) kinase inhibitor, shows promising antitumour activities in various cancers including lung cancer. Here, we explored the effects and the underlying molecular mechanism of CED on non-small-cell lung cancer (NSCLC) cell line A549 cells in vitro. Our results show that CED could inhibit A549 cell proliferation and cloning formation. Meanwhile, G1 phase cell cycle arrest was also found, as featured by the increased proportion of G1 phase cells as well as the reduction of G1 phase relative proteins CDK4/cyclin D1 and CDK2/cyclin E. Moreover, the ratio of LC3-II/LC3-I was elevated significantly in CED-treated groups compared with the controls. Furthermore, the expression of p-Akt, p-P38, p-Erk1/2, and p-mTOR proteins was decreased obviously in the treatment groups. These results suggest that CED could induce apoptosis and G1 phase cell cycle arrest in A549 cells. Meanwhile, CED may induce autophagy through MAPK/Erk1/2 and Akt/mTOR signal pathway in A549 cells.

## 1. Introduction

Lung cancer is one of the most common malignancies and ranks the 1st in both incidence and mortality, with approximately 2.09 million new cases and 1.76 million deaths in the world in 2018 according to the Internal Agency for Research on Cancer. NSCLC accounts for approximately 80-85% of all lung cancers, and the 5-year survival rate for NSCLC patients was less than 15% currently [1, 2]. Approximately 80% patients with NSCLC develop metastases within 2 years after the diagnosis of NSCLC. Early diagnosis of lung cancer before clinical symptoms is an effective means to reduce cancer mortality, and the use of low-dose CT scans contributes to a 20% reduction in lung cancer mortality according to National Lung Screening Trial [3]. Unfortunately, over 60% of patients are diagnosed with lung cancer in the advanced stages [4]. In recent years, VEGFR inhibitors such as Ramucirumab have improved the prognosis of patients with NSCLC [5], suggesting that VEGFR inhibitor is a new strategy for NSCLC treatment.

The VEGF pathway plays an essential role in angiogenesis. It was reported that high levels of VEGF were correlated with NSCLC. Meanwhile, VEGFR is upregulated in metastatic prostate cancer, and VEGF-D, VEGFR2, and VEGFR3 are related to advanced-stage prostate disease [6]. Moreover, high VEGF-C expression predicts an adverse prognosis in pediatric and adult acute myeloid leukemia [7]. VEGF signaling affects tumour progress mainly in three ways. First, VEGF ligands interact with VEGFR tyrosine kinases on the surface of endothelial cells, which we refer to as “canonical” VEGF signaling. It appears to be a common form to induce cell proliferation, migration, and vascular morphogenesis [8]. HIF-1 expression is elevated under hypoxia condition, and elevated HIF-1 subsequently promotes autocrine VEGF in tumour cells. The secreted VEGF binds to specific receptors on the surface of tumour cells, such as neuropilin-1 and Flt-1, and affects cell survival and migration through the activation of downstream signaling pathways such as PI3K/Akt signaling [9–11]. This is the second pathway by which VEGF maintains its own development, also known as the autocrine pathway. Meanwhile, a line of evidence presented that VEGFR tyrosine kinases can be activated in an independent manner through Src kinases or alternative “ligands” such as Gal3 and Gall, and this is considered to be “noncanonical” VEGF signaling [12–14]. Therefore, at least 3 strategies, including VEGF inhibitor, VEGFR inhibitor, and VEGFR tyrosine kinase inhibitor, have been applied to inhibit the VEGF signal pathway.

CED, a highly potent VEGFR1, VEGFR2, and VEGFR3 tyrosine kinase inhibitor, has shown promising antitumour activity in prostate cancer [15], glioblastoma [16], metastatic breast cancer [17], and renal cell carcinoma [18] *in vitro*, *in vivo*, and in clinical trials. MFOLFOX6 in combination with CED can prolong progression-free survival in patients with metastatic colorectal cancer [19]. There is evidence that CED-cisplatin cotreatment can reduce tumour dissemination and prolong the survival of mice bearing ovarian cancer [19]. Besides, antitumour activities of CED in lung cancer rely on the inhibitory activity against the VEGFR family and the platelet-derived growth factor receptor- (PDGFR-) related kinases c-Kit, PDGFR-*α*, and PDGFR-*β* [21]. Many studies have shown that CED has beneficial effects on lung cancer [21, 22]. Devery et al. have found that VEGFR2 protein and mRNA are elevated in 9/25 NSCLC cell lines and is closely correlated with cancer angiogenesis. VEGFR2 is expressed in both squamous cells and adenocarcinoma cells, but not in normal lung epithelial cells [22]. Meanwhile, administration of CED can rapidly reduce the perfusion of Calu-3 cancers (stromal vessel phenotype), leading to acute hypoxia in lung cancer [23]. Additionally, low baseline VEGFR2 and VEGFR3 were predictive for both overall survival and progression-free survival in CED-treated NSCLC patients. Furthermore, combined MEK inhibitor selumetinib with VEGFR inhibitor CED in lung cancer results in decreased cell proliferation, metastasis, and angiogenesis. However, the mechanism remains to be explored [24]. In the present study, we aimed to validate the underlying molecular mechanisms of CED in A549 cells.

## 2. Materials and Methods

### 2.1. Materials

The human NSCLC cell line A549 (p53 wild-type) was provided by the Cell Bank of Chinese Academy of Sciences (Shanghai, China). CED (purity > 99.58%) was purchased from MedChemExpress (New Jersey, USA). CED was dissolved in dimethyl sulfoxide (DMSO) at a concentration of 10 mM and stored at -80°C.

Fetal bovine serum (FBS) was purchased from Gibco (Carlsbad, CA, USA). Mouse anti-microtubule-associated protein B-light chain 3 (LC3B) was purchased from Cell Signaling Technology (Billerica, USA). Rabbit anti-GAPDH, mouse anti-mTOR, and rabbit anti-p-mTOR were purchased from Invitrogen (California, USA). Rabbit anti-VEGFR2, rabbit anti-VEGFR3, rabbit anti-P38, rabbit anti-p-P38, rabbit anti-Erk1/2, and rabbit anti-p-Erk1/2 were acquired from GeneTex (Taiwan, China). Rabbit Anti-CDK4, rabbit anti-cyclin D1, rabbit anti-CDK2, and rabbit anti-cyclin E were purchased from Abcam (Cambridge, England).

Cell counting kit-8 (CCK-8) was obtained from Yeasen Biotech (Shanghai, China). Antifade mounting medium with 4′,6-diamidino-2-phenylindole was obtained from Vector Laboratories (Shanghai, China). Annexin V-FITC/PI double staining was purchased from Becton, Dickinson and Company (New Jersey, USA).

### 2.2. Cell Culture

The human NSCLC cell line A549 was maintained in RPMI-1640 medium, which is supplemented with 10% FBS. Cells were incubated at 37°C in a 5% CO_2_ humid environment. The cells were replaced with fresh medium every other day and plated at an appropriate density according to experimental needs.

### 2.3. Cell Viability Assay

CCK-8 was used to determine the proper concentrations of CED in A549 cells. The cells were incubated in 96-well plates at a density of 6000 per well with 200 *μ*l complete medium. After 24 h, cells were treated with an increasing dose of CED (0 *μ*M, 3 *μ*M, 6 *μ*M, 9 *μ*M, 12 *μ*M, and 15 *μ*M) for 24 h, 48 h, and 72 h, respectively. 0.15% DMSO, the same DMSO concentration as the 15 *μ*M group, was used to test the toxic effects of DMSO on the cells. Subsequently, CCK-8 solution (20 *μ*l) was added to the cell culture sample and further incubated for 1 h at 37°C. The absorbance of each well was measured at 450 nm by using a microplate reader (Tecan infinite 200 M, Männedorf, Switzerland).

### 2.4. Colony-Forming Assay

A colony-forming assay was applied to detect cell survival activity in vitro. Briefly, cells were seeded onto the 60 mm dish at proper density with 2 ml culture media 24 h before treatment with different concentrations of CED (0 *μ*M, 3 *μ*M, 6 *μ*M, and 9 *μ*M). After incubating with CED for 48 h, the cells are digested and resuspended and then were incubated in a 60 mm dish for 10~14 days with drug-free medium. Colonies were fixed with 4% paraformaldehyde for 20 min followed by dyeing with 1% crystal violet for 15 min at room temperature. The colonies consisting of more than 50 cells were counted under a light microscope.

### 2.5. Cell Apoptosis Assay

The cells treated with CED for 48 h were digested with EDTA-free trypsin. Then, digested cells were washed with PBS followed by resuspending in 150 *μ*l binding buffer. 5 *μ*l propidium iodide (PI) and 5 *μ*l Annexin V-FITC were added and incubated for 15 min at room temperature under dark condition. Cell apoptosis at each stage was detected on a flow cytometer FlowSight (Washington, USA) and analyzed by IDEAS Application v6.0 software. Each sample was collected at least 10,000 events.

### 2.6. Cell Cycle Assay

Cells were treated with different concentrations of CED for 48 h and then were digested with trypsin followed by fixing with 75% ethanol at -20°C. After overnight incubation, cells were resuspended and rehydrated in PBS for 15 min. Subsequently, cells were resuspended in 150 *μ*l DNA Staining Solution for 20 min under dark condition. The cell cycle was detected by flow cytometry and analyzed by FlowJo-V10 software. At least 20,000 events were examined per sample.

### 2.7. Immunofluorescence

A549 cells were seeded on the slices in 35 mm dishes and incubated overnight. Then, cells were treated with different concentrations of CED for 48 h. Subsequently, slices were fixed with 4% paraformaldehyde after washing with phosphate-buffered saline (PBS) for 3 times. 0.25% TritonX-100 was added to permeabilize the cells for 15 min. After washing with PBS for 3 times, the cells were blocked with 5% BSA for 70 min at room temperature (RT). After blocking, the cells were incubated with primary antibody against LC3B at 4°C overnight and then incubated with secondary antibody at room temperature for 1.5 h. Finally, cells were counterstained with DAPI for 5 min in the dark. Images were captured using a laser scanning confocal microscope (Carl Zeiss AG, Germany).

### 2.8. Western Blot Analysis

Cells were incubated in different concentrations of CED. After 48 h, the cells were washed with PBS and then lysed with Lysis Buffer (RIPA: PMSF = 100 : 1) on ice for 30 min. A BCA protein assay kit was used to detect the concentration of protein. The total proteins were separated by sodium dodecyl sulfate-polyacrylamide gel electrophoresis at 20 *μ*g per gel lane, followed by transfer to a PVDF membrane. The membrane was blocked for 1 h, followed by incubation with primary antibodies against CDK4, cyclin D1, CDK2, cyclin E, LC3B, Akt, p-Akt, P38, p-P38, Erk1/2, p-Erk1/2, mTOR, and p-mTOR overnight at 4°C. Then, the membranes were washed and then incubated with secondary antibodies. Density values of target protein bands were detected by using an ECL kit from Yeasen Biotech (Shanghai, China). GAPDH was used to normalize the results.

### 2.9. Statistical Analysis

The experiments were totally repeated for three times, and data were presented as the mean ± SD. Statistical significance was evaluated using one-way analysis of variance (ANOVA) with the LSD (L) post hoc test in SPSS 16.0 (IBM SPSS Statistics 20.0, Armonk, NY). Origin8.0 (Origin Lab, Northampton, MA) software was used to plot the figures.

## 3. Results

### 3.1. CED Inhibited A549 Cell Proliferation

**Figure 1:**
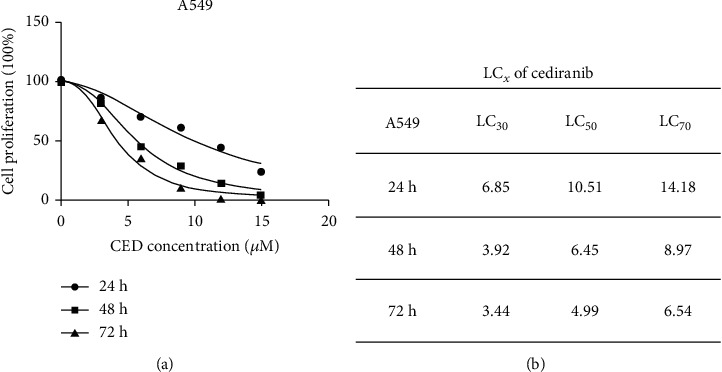
CED significantly inhibited A549 cell viability. (a) CCK-8 assay revealed the viability of A549 cells affected by CED. (b) LC_30_, LC_50_, and LC_70_ of CED in A549 cells for 24 h, 48 h, and 72 h. The data represent the mean ± SD.

In order to investigate the effect of CED on cell viability, the cells were treated with different concentrations of CED for 24 h, 48 h, and 72 h, respectively. The CCK-8 assay showed that CED could markedly inhibit the growth of A549 cells in a concentration- and time-dependent manner. The Bliss method was used to calculate the lethal concentrations (LC_*x*_) of CED. Our data showed that LC_30_, LC_50_, and LC_70_ of CED in A549 cells for 48 h were 3.92 *μ*M, 6.45 *μ*M, and 8.97 *μ*M, respectively (Figure 1). The cell proliferation rate of A549 cells after 24 h of CED treatment was significantly higher than that of 48 h. Therefore, A549 cells were treated with CED for 48 h for the subsequent experiments. The following concentrations 0 *μ*M, 3 *μ*M, 6 *μ*M, and 9 *μ*M which can cover LC_30_, LC_50_, and LC_70_ of CED were selected. 0.15% DMSO had no toxic effect on cells, so the subsequent experiments did not examine the effect of DMSO on A549 cells alone.

### 3.2. CED Inhibited A549 Cell Colony Formation

**Figure 2:**
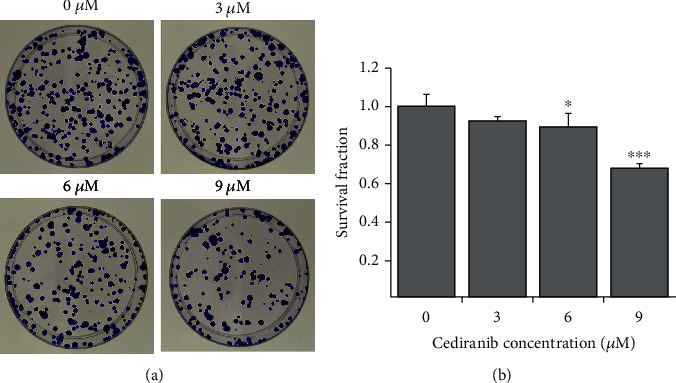
CED treatment could inhibit A549 cell colony formation. (a) Representative pictures of colony-forming potential of A549 cells in different treatment groups. (b) Cell survival fraction bar chart was performed with cell clone formation assay. Cells were treated with the indicated concentration of CED for 48 h and then plated for survival. All experiments were performed in triplicate. The data represent the mean ± SD. ^∗^*p* < 0.05 and ^∗∗∗^*p* < 0.001 (vs. control group).

We observed that the ability of cell clone formation decreased with the increase of drug concentration. The clone survival rate of the 3 *μ*M-, 6 *μ*M-, and 9 *μ*M-treated groups was 92.4%, 89.6%, and 67.7%, respectively. Cell viability reduced approximately 10.4% (*p* < 0.05) and 32.34% (*p* < 0.001) after treatment with 6 *μ*M and 9 *μ*M CED for 48 h compared with the controls (Figure 2). However, there was no statistical significance in the 3 *μ*M-treated group compared with the controls.

### 3.3. CED Induced A549 Cell Apoptosis

**Figure 3:**
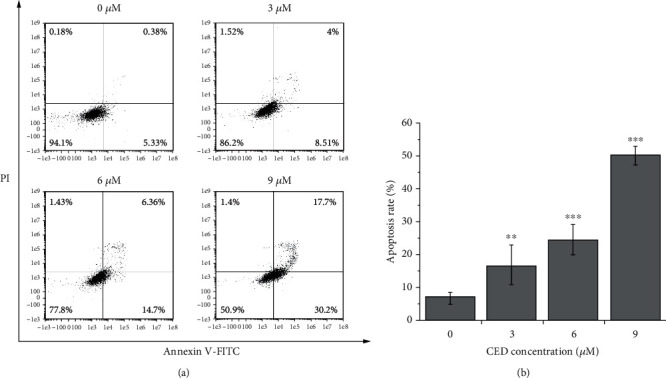
CED treatment for 48 h induced cell apoptosis in A549 cells. (a) Cell apoptosis assay was investigated with flow cytometry. (b) Percentages of apoptotic rate and cell population were calculated. All experiments were performed in triplicate. All data are representative of three independent experiments. ^∗∗^*p* < 0.01 and ^∗∗∗^*p* < 0.001 compared with controls.

To further explore whether the decrease of cell survival rate induced by CED is implicated in apoptosis, an Annexin V-FITC/PI double staining assay was used to detect the apoptosis of A549 cells. As shown in Figure 3, our results indicated that cell apoptosis was significantly induced after 48 h of CED treatment. The apoptosis rate was positively correlated with the dose. The apoptosis rates were 12.51%, 21.06%, and 47.9%, respectively, after 3 *μ*M, 6 *μ*M, and 9 *μ*M CED treatment, compared with 7.59% in the control group. Moreover, cells treated with 3 *μ*M, 6 *μ*M, and 9 *μ*M CED induced 2.34-fold (*p* < 0.01), 3.44-fold (*p* < 0.001), and 7.04-fold (*p* < 0.001) apoptosis compared with the controls.

### 3.4. CED Induced A549 Cell Cycle Arrest in G1 Phase

**Figure 4:**
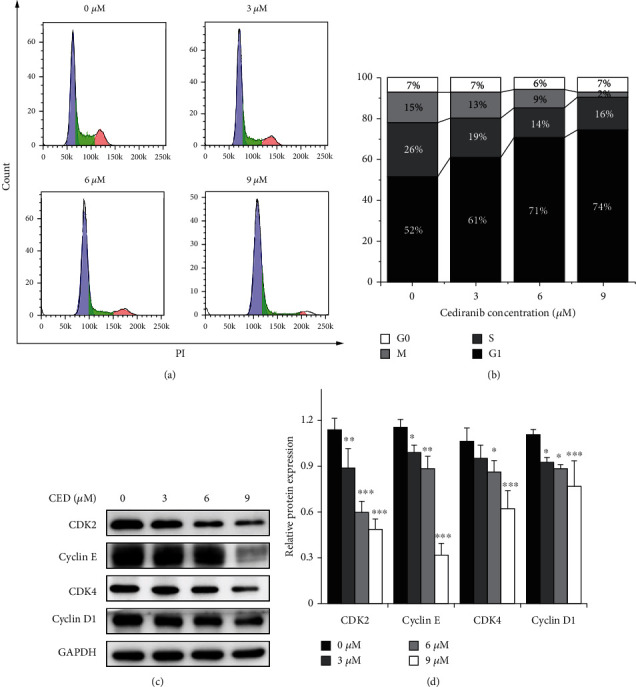
CED treatment induced G1 phase arrest in A549 cells after 48 h. (a) Cell cycle distribution in A549 cells pretreated with the indicated concentration of CED at the time point of 48 h. DNA content of cells stained with PI was detected using flow cytometry. (b) The percentage of the cell population in each phase was quantitatively analyzed after exposure to the indicated CED for 48 h. (c) After CED treatment, G1 phase relative proteins CDK2, cyclin E, CDK4, and cyclin D1 were detected by western blot analysis. (d) Quantitative analysis of CDK2, cyclin E, CDK4, and cyclin D1 proteins was represented by column graphs. Bars represented the mean ± SD from three independent experiments. ^∗^*p* < 0.05, ^∗∗^*p* < 0.01, and ^∗∗∗^*p* < 0.001 (vs. control group).

Cell cycle arrest was analyzed by flow cytometry after treatment with CED for 48 h. Compared with the control group, an increasing percentage of G1 phase cells was observed at all doses (Figures 4(a) and 4(b)), accompanied by a decrease in the percentage of S and G2/M phase cells. The proportion of G1 phase cells in the 3 *μ*M, 6 *μ*M, and 9 *μ*M groups were 61%, 71%, and 74%, respectively, and were observably higher than that of the controls (52%). The positive correlation between G1 phase retardation and concentration suggests that CED may suppress the proliferation of A549 cells by inducing G1 phase cell cycle arrest. Furthermore, CED significantly affected the expression of G1 phase-related proteins CDK2, cyclin E, CDK4, and cyclin D1. As shown in Figures 4(c) and 4(d), CED could remarkably inhibit the growth of A549 cells in a concentration-dependent manner. The expression of cell cycle-related proteins was significantly decreased in the 6 *μ*M- and 9 *μ*M CED-treated groups. 3 *μ*M CED treatment can also slightly decrease cell cycle-related proteins cyclin E, cyclin D1, and CDK2. However, there was no significant difference in the expression of CDK4 protein compared with the control group.

### 3.5. CED Induced A549 Cell Autophagy

**Figure 5:**
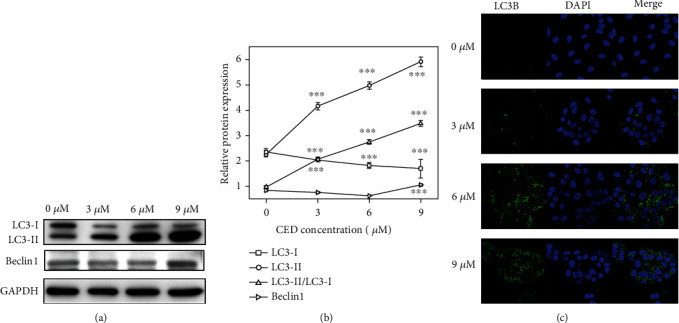
CED treatment activated autophagy in A549 cells. (a) A549 cells from indicated treatment groups were incubated for 48 h, and autophagy relative proteins (LC3B and Beclin1) were detected using western blot. (b) Quantitative analysis of Beclin1, LC3-I, and LC3-II proteins in A549 cells by western blot analysis. (c) The localization of LC3B protein in A549 cells supplemented with CED was determined by fluorescent microscopy. All data are representative of three independent experiments. The data are expressed as the mean ± SD. ^∗∗∗^*p* < 0.001 (vs. control group).

Under physiological conditions, autophagy is at a low level. When the body is under stress, cells degrade and recycle their intracellular components by inducing autophagy [25]. We tested whether CED could induce autophagy in A549 cells using a western blot assay and fluorescence microscopy. Rat microtubule-associated protein light chain 3 (LC3), a homologue of yeast Atg8, is an essential component of autophagy. The transition from LC3-I to LC-3II, which relies on the Atg7/Atg3 ubiquitin-like system, has been recognized as a marker of autophagy [26]. Therefore, protein levels of LC3-I and LC3-II in CED-treated cells were detected using GAPDH as a control. The expression of Beclin1, an autophagy marker protein, was also detected. Our data shows that Beclin1 was upregulated in the 9 *μ*M group. Compared with the controls, CED treatment strongly increased LC3-II and decreased LC3-I proteins in a dose-dependent manner (Figures 5(a) and 5(b)). Additionally, an increase in the ratio of LC3-II/LC3-I was clearly observed (2.14-fold (*p* < 0.001), 2.86-fold (*p* < 0.001), and 3.64-fold (*p* < 0.001), respectively, for 3 *μ*M, 6 *μ*M, and 9 *μ*M CED treatment *vs*. the controls). Moreover, confocal microscopic (LSCM) biomedical images showed that the application of CED dramatically accumulated LC3B puncta in 6 *μ*M and 9 *μ*M groups while LC3B puncta in the 3 *μ*M group seems weak (Figure 5(c)).

### 3.6. CED Suppressed Akt/mTOR and MAPK Signal Pathway

**Figure 6:**
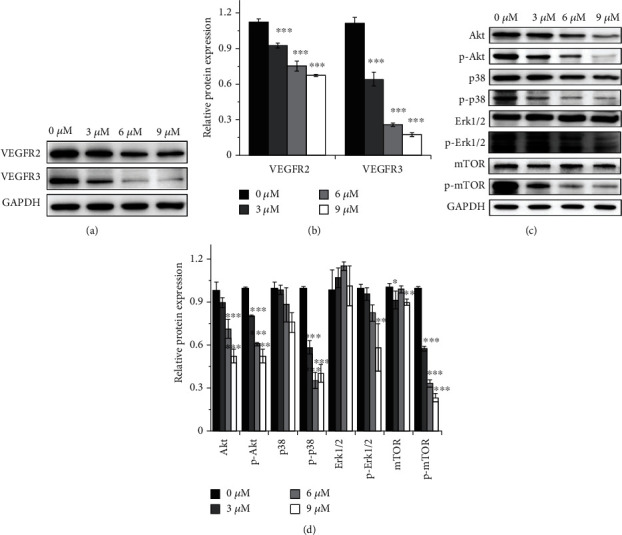
Effect of CED on proteins involved in VEGFR/Akt/mTOR and VEGFR/MAPK pathway in A549 cells after 48 h treatment. (a, c) Representative western blot images. (b, d) Quantitative analysis of the expression of VEGFR2, VEGFR3, Akt, p-Akt, p-38, p-p38, Erk, p-Erk, mTOR, and p-mTOR, as represented by column graphs. GAPDH was used as a control. All data are representative of three independent experiments. The data are expressed as the mean ± SD. ^∗^*p* < 0.05, ^∗∗^*p* < 0.01, and ^∗∗∗^*p* < 0.001 (vs. control group).

In order to elucidate the role of CED in cancer therapies, we examined the representative proteins in VEGFR, Akt/mTOR, and MAPK signal pathways by western blot. Our data demonstrated that the expression of VEGFR2 and VEGFR3 was consistently downregulated after CED treatment (Figures 6(a) and 6(b)). Phosphorylated Akt (p-Akt), phosphorylated p38 (p-p38), and phosphorylated mTOR (p-mTOR) were also inhibited by CED in all doses while phosphorylated Erk1/2 (p-Erk1/2) was only significantly decreased in the 9 *μ*M group (Figures 6(c) and 6(d)). In addition, p38, Erk1/2, and mTOR had no change in all groups. Meanwhile, we found that CED can reduce Akt expression in the 6 *μ*M group and the 9 *μ*M group. These data suggested that CED may induce autophagy and G1 phase cell cycle arrest through suppressing MAPK and Akt/mTOR signal pathways. However, whether the inhibition of MAPK and Akt/mTOR signal pathways is related to the reduction of VEGFR2 and VEGFR3 needs further study.

## 4. Discussion

**Figure 7:**
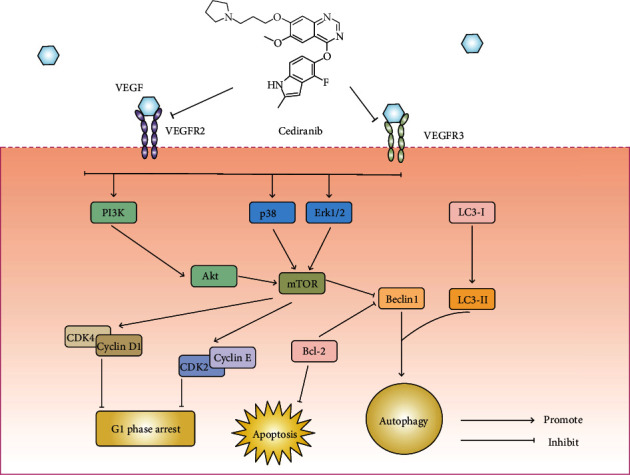
Schematic depicting the effect of CED on cell survival and the underlying mechanism. CED inhibits the expression of VEGFR2/3 and suppresses MAPK/Erk1/2 and Akt/mTOR signaling pathways in A549 cells. This eventually leads to apoptosis, G1 phase cell cycle arrest, and autophagy, as featured by the decrease of G1 phase relative proteins CDK4/cyclin D1 and CDK2/cyclin E and the increase of autophagy relative proteins LC3-II and Beclin1.

Lung cancer is one of the most common malignant tumours which severely endanger human health and life with the highest morbidity and incidence. Drug resistance remains the obstacle to lung cancer treatment. Therapies targeting VEGFR signaling, such as Apatinib, Ramucirumab, and Axitinib, show promising effects on suppressing the proliferation and angiogenesis of gastric cancer, osteosarcoma, head and neck cancer, and NSCLC [27–30]. CED is a VEGFR tyrosine kinase inhibitor and shows anticancer potential against various types of tumours [31]. Our findings demonstrate that CED inhibited cell proliferation and clonal formation while inducing apoptosis, G1 phase cell cycle arrest, and autophagy in A549 cells.  Figure 7 systematically depicts the effect of CED on cell survival and the underlying mechanism.

Apoptosis is a kind of programmed cell death, occurring under either physiological or pathological conditions such as normal cell turnover, immune reaction, and radiation/chemical-induced cell death [32]. Inducing cancer cell apoptosis is the ultimate goal of cancer therapies. The mechanism of apoptosis is divided into two main pathways: the intrinsic pathway (mitochondrial pathway) and the extrinsic pathway (death receptor pathway). In addition, there is an additional pathway that involves T-cell mediated cytotoxicity and perforin-granzyme-dependent cell killing [32, 33]. Annexin V/PI double staining indicated that CED can strongly induce apoptosis in A549 cells. However, potential mechanisms of apoptosis induction need to be further explored.

Cell cycle progression, which provides an opportunity for damage repair and stopping their transmission to the daughter cells, is principally modulated through cyclin-dependent kinase (CDKs) [35]. Cyclins form complexes with their specificity CDKs and act as their regulatory subunit [35]. Reversing the repressing transcription of many genes, which are essential for cells to exit from the G1 phase, is required for the cyclin D/CDK4/6 complex. CDK2 together with cyclin E governs the G1/S transition and the progression of the S phase [37].

We used flow cytometry to detect the cell cycle, and the results showed that CED could increase the proportion of G1 phase cells while decreasing the proportion of S and G2/M phase cells. The later protein quantification also discovered the decreased expression of CDK4, cyclin D, CDK2, and cyclin E. Our data indicated that CED could inhibit the transition of G1 to S phase and induce G1 phase cell cycle arrest in A549 cells.

Autophagy is an innate self-regulating process that removes dysfunctional or superfluous proteins and organelles with the help of digestive enzymes in lysosomes [38]. LC3-II and Beclin1 are representative markers of autophagy. The elevated LC3-II and Beclin1 in treated groups were confirmed by western blot, suggesting that CED could induce autophagy in A549 cells.

It is worth noting that autophagy is a double-edged sword. On one hand, autophagy helps cells adapt to changing living conditions and recycling cytoplasm. On the other hand, autophagy or autophagy-relevant proteins may lead to apoptosis, necrosis, pyroptosis, or “autophagic-cell death” once autophagy degrades the cytoplasm excessively [39]. Meng has found that autophagy inhibitor in combination with VEGFR2 inhibitor Apatinib can further inhibit papillary thyroid carcinoma cell proliferation and induce PARP/Bcl-2-mediated apoptosis [40]. Gefitinib is an EGFR-TKI. Evidence suggests that autophagy contributed to Gefitinib resistance in NSCLC cells [41]. Therefore, the role of autophagy in tumour therapy is worth further exploring.

In order to meet the need for rapid proliferation, tumour cells promote angiogenesis by secreting proangiogenic factors such as PDGF, TGF, and VEGF family. Among them, the VEGF family is the most important factor for angiogenesis. After activating by VEGF, VEGFR dimerizes themselves and then recruits specific downstream signal transduction mediators to trigger signal transduction, following by the activation of downstream signaling pathways such as the PI3K/Akt/mTOR signal pathway and MAPK signal pathway [42, 43]. Earlier researches have reported that mTOR is a common effect factor of Akt and MAPK signal pathway and suppressed autophagic activity by combining with the positive autophagy regulator Atg1/ULK1 complexes [44]. Beclin1 is a key molecule that initiates nucleation of autophagy isolation vesicles. Under normal conditions, Beclin1 binds to Bcl-2 through the BH3 domain, and they are inactivated by each other. Ser/Thr kinases such as Akt could regulate autophagy and apoptosis by phosphorylating Beclin1 within its BH3, which reduces its inhibitory interaction with Bcl-2 or Bcl-XL [44].

According to our data, CED treatment results in the activation of autophagy flux through the Akt and MAPK signal pathway, as featured by the reduction of p-Akt, p-mTOR, p-38, and p-Erk1/2 and the accumulation of LC3-II and Beclin1. CED relieved the inhibition effect of mTOR on autophagy.

Simultaneously, mTOR was reported to act as a positive regulator of the cell cycle. Inactivation of mTOR can induce G0/G1 phase cell cycle arrest by downregulating S6K1 and 4E-BP1 [45]. Meanwhile, previous studies have found that microRNA-646 [46] and microRNA-99a [47] could induce G1 phase cell cycle arrest by downregulating mTOR signals, while overexpression of mTOR reversed the inhibitory effect of microRNA-646 on cell cycle progression. The role of mTOR in CED-induced G1 phase cell cycle arrest is complicated, and extensive exploration is needed.

## 5. Conclusion

In conclusion, this study verified that CED effectively suppressed cell viability and induced apoptosis, autophagy, and G1 phase cell cycle arrest in A549 cells. Meanwhile, CED may induce autophagy and G1 phase cell cycle arrest through the Akt/mTOR signal pathway and MAPK pathway.

## Data Availability

The data used to support the findings of this study are available from the corresponding authors upon request.
